# Anti-Diabetic Effects of *Allium hookeri* Extracts Prepared by Different Methods in Type 2 C57BL/J-*db/db* Mice

**DOI:** 10.3390/ph15040486

**Published:** 2022-04-17

**Authors:** Ji-Hye Choi, Si-Hyun Kim, Eun-Byeol Lee, Ji-Su Kim, Ji-Eeun Jung, Un-Yul Jeong, Ju-Hui Kim, Hwan-Hee Jang, Shin-Young Park, Gi-Chang Kim, Jung-Hyun Lim, Sung-Hyen Lee

**Affiliations:** 1 National Institute of Agricultural Sciences, Rural Development Administration, Wanju 55365, Korea; jyyye@jbnu.ac.kr (J.-H.C.); sihyun4248@gmail.com (S.-H.K.); leeeb1029@korea.kr (E.-B.L.); ver0218@korea.kr (J.-S.K.); jjempm@korea.kr (J.-E.J.); narcism077@jbnu.ac.kr (U.-Y.J.); juhui5466@korea.kr (J.-H.K.); rapture19@korea.kr (H.-H.J.); soyeonj@korea.kr (S.-Y.P.); recall@korea.kr (G.-C.K.); jhlim0531@korea.kr (J.-H.L.); 2 Department of Laboratory Animal Medicine, College of Veterinary, Jeonbuk National University, Iksan 54596, Korea

**Keywords:** *Allium hookeri*, diabetes, glucose, lipid, immunity

## Abstract

This study was conducted to evaluate whether *Allium hookeri* can control diabetic symptoms. Aqueous extract (AE1: 100 mg/kg BW, AE2: 200 mg/kg BW) and ethanol extract (EE1: 100 mg/kg BW, EE2: 200 mg/kg BW) of *A. hookeri* were orally administrated to diabetic mice (C57BL/J-*db/db*) for 8 weeks. The negative (NC) and the positive (PC) control groups were treated with 0.9% saline and metformin (150 mg/kg BW), respectively. Glucose and lipid profile (triglyceride, total cholesterol (TC), LDL-C, and HDL-C) as biochemical parameters, toxicological factors such as liver/kidney functional parameters (ALT, AST, BUN, and Cr), and NK cell activity in blood were measured. Oral glucose tolerance test (OGTT) and histopathological examination were also conducted. Compared with the NC group, AE and EE decreased blood glucose, HbA1c, area under the curve (AUC) during OGTT, and leptin levels while increasing adiponectin levels. Serum lipid profiles and toxicological factors levels were reduced by the *A. hookeri* extract. Interestingly, HDL-C, glomerular mesangial expansion score in the kidney, and NK cell activity were effectively controlled in EE groups. Based on the results, EE is considered to be more effective in reducing high blood glucose, lipid profile, and related factor levels than AE, and is comparable to metformin in some biomarkers. It can be presumed that EE can more effectively control the major anomalies in the diabetic model than AE, and it may be used to prevent diabetic symptoms without toxicity in the Type 2 diabetic model.

## 1. Introduction

Diabetes mellitus was described in ancient scripts and is recognized as a serious illness. Human health is being increasingly affected by the rising numbers of people with diabetes mellitus [1]. Diabetes mellitus is a chronic illness characterized by elevated levels of blood glucose, accompanied by the disturbed metabolism of fats and proteins. Blood glucose rises because it cannot be metabolized in the cells, due to a lack of insulin production by the pancreas or the inability of the cells to effectively use the insulin that is being produced [2]. There are two major types of diabetes: the pancreas does not produce insulin in Type 1, whereas in Type 2 the body cells are resistant to the action of insulin that is being produced and over time the production of insulin progressively decreases. Type 2 diabetes mellitus (T2D), which accounts for 90% of diabetic patients, is closely correlated with the increase in the obese population and is characterized by insulin resistance [3]. T2D is a metabolic disorder characterized by hyperglycemia and is one of the most common chronic diseases worldwide [4]. The primary causes of T2D are insulin resistance, an insulin secretion defect due to beta-cell failure, and hepatic excess glucose production [5,6]. It has been also reported that T2D and obesity develop inflammation due to insulin resistance [7].

The increased elevation of aminotransferase (ALT) and aspartate aminotransferase (AST). ALT and AST indicates the impaired metabolic function of the liver and the increased risk of hepatitis, cirrhosis, and T2D [8]. These factors are frequently used as a primary marker to check hepatic function. The treatment of *Saccharina japonica* inhibited the excess elevation of the AST and ALT, which were higher in diabetic mice than in non-diabetic mice and treated the toxicity of the diabetic mice [9]. Similarly, several earlier works showed that plant extracts such as *Sida cordata*, *Vitis vinifera*, and *Fagonia olivieri* prevent liver damage, which leads to the decreased elevation of the AST and ALT in diabetic mice [10,11,12]. Blood urea nitrogen (BUN) and creatinine (Cr) levels were found to be significantly higher in the diabetic mice than in non-diabetic mice [9]. The higher elevation of BUN and Cr indicates kidney damage in diabetic mice in contrast to non-diabetic mice [10]. Serum urea and Cr are biomarkers that can serve as predictor tests for assessing kidney function (nephropathy) in diabetic patients. A correlation of serum Cr and urea with glycemic index and the duration of diabetes mellitus in both Type 1 and Type 2 was reported [13]. The treatment of *S. japonica* retained the normal elevation of all tested serum markers including insulin and lipid profile by preventing histopathological injuries in the diabetic mice [10].

T2D drives immune dysfunction, infection development, and sepsis mortality [14,15]. Individuals with T2D have abnormal host responses, including disorders of humoral immunity, and defects in neutrophil function and T cell response. [16]. So, the role of the immune system is more important in T2D than in normal individuals [17].

It has been reported that the oral hypoglycemic agents currently used for the treatment of diabetes can delay glucose absorption in the digestive tract depending on the mechanism of action. Among them, metformin has been considered as an insulin sensitizer because it lowers blood sugar without increasing insulin secretion [18]. Metformin has shown beneficial effects in T2D, including weight loss, improved lipid profile, and improved endothelial function. Antidiabetic drugs are being developed and used, but the drugs may cause side effects such as hypoglycemia, vomiting, and liver function damage. Research on natural materials that can reduce these side effects and control blood sugar has been continued [19,20,21,22]. Eslami et al. reported on the interaction between dietary patterns and immunometabolism as a new frontier for diabetes mellitus and related disorders [23] and Shabab et al. introduced some traditional herbal drugs as alternative medicines for diabetes mellitus [24,25,26].

*Allium hookeri* (AH) is a plant of the genus *Allium*, which includes onions, green onions, garlic, and chives, and contains special amino acids such as *S*-Allyl-l-cysteinsulfoxide (ACSO), alliin, cycloalliin, and natural compounds such as volatile sulfur compound saponin [27]. It has been reported that the sulfur-containing compounds of allicin and alkyl thiosulfinate of AH have various physiological activities, such as lowering blood sugar and adipogenesis in diabetic models [25,28,29,30]. However, there is currently no comparative study on the glycemic control and anti-diabetic efficacy of AH extracts prepared by different methods, such as water and ethanol extraction as a solvent. Thus, the two kinds of extracts were administered to the T2D model, and their anti-diabetic effects on glucose, hepatic toxicity, lipid profile, and immunity, which are major disorders in T2D, were evaluated.

## 2. Results

### 2.1. Concentration of Cycloalliin

**Figure 1:**
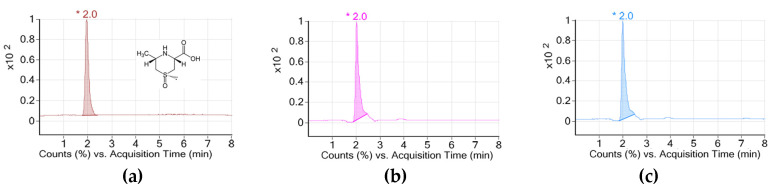
Chromatograms of cycloalliin in (**a**) standard, (**b**) aqueous, and (**c**) ethanol extracts of *A. hookeri* analyzed by LC/MS.

Figure 1 shows the contents of cycloalliin (C_6_H_11_NO_3_S) in aqueous (AE) and ethanol (EE) extracts of *A. hookeri*. Each concentration was 2.94 and 3.11 mg/g, respectively. No significant difference was found in cycloalliin concentrations between two extracts.

### 2.2. Effects of A. hookeri Extracts on Body and Organ/Tissue Weights

Table 1 shows the body and tissue weights of diabetic mice, which were measured in this experiment. The initial and final body weights of the diabetic mice were significantly higher than those of the Control mice. However, there was no significant difference in the initial and final body weights of the diabetic mice treated with or without any AH extract. The weights (% of BW) of liver, kidneys and epididymal fat were significantly higher in the NC group compared with those in the Control group. However, the organ weights decreased in AH-administered groups in a dose-dependent manner, and epididymal fat weights were significantly lower in the mice treated with AE2 and EE2 (AH 200 mg/kg BW) than those in the NC group. AH also increased the spleen weights of the mice in a dose-dependent manner.

### 2.3. Effects of A. hookeri Extracts on Fasting Blood Glucose

**Figure 2:**
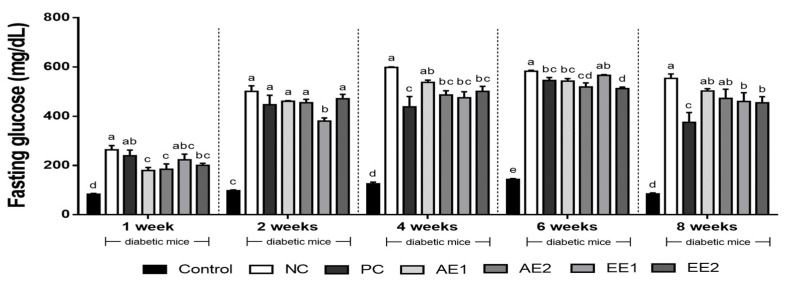
Effects of *A. hookeri* extracts on fasting blood glucose levels in the diabetic mice. Control: non-diabetic mice; NC: negative control; PC: positive control, metformin 150 mg/kg BW; AE1: aqueous extract 100 mg/kg BW; AE2: aqueous extract 200 mg/kg BW; EE1: ethanol extract 100 mg/kg BW; EE2: ethanol extract 200 mg/kg BW. AE and EE groups were treated with *A. hookeri* extracts for 8 weeks. Data are presented as the mean ± SEM (n = 8). ^a–e^ Mean values with different letters are significantly different at *p* < 0.05.

In the first week of the experiment, the significantly highest blood glucose level was observed in the NC group rather than in the Control group, while the tendency to decrease in the *A. hookeri* extract-administered groups was detected (Figure 2). After 2 weeks of the administration of the AH extracts, a significant decrease of 14% in the fasting blood glucose was found only in the EE1 group compared with the NC group. The AE2 and the EE groups showed significantly decreased blood glucose levels compared with the NC group in the 4th week. After another 2 weeks (the 6th week), the fasting blood glucose levels of the AE2 and EE2 groups significantly decreased by over 12% compared with the NC group (*p* < 0.05), and the lowest value was found in the EE2 group. Interestingly, the EE1 and EE2 groups, which were administered the ethanol extract, showed 12% and 13% decreases in the blood glucose levels compared with the NC group, respectively, while there was no significant difference between the NC and AE groups treated with the hot water extract by the 8th week of this experiment. Thus, the ethanol extract of *A. hookeri* could be more effective controlling the fasting blood glucose levels than the aqueous extract of *A. hookeri* in diabetic mice.

### 2.4. Effects of A. hookeri Extracts on Oral Glucose Tolerance and AUC

**Figure 3:**
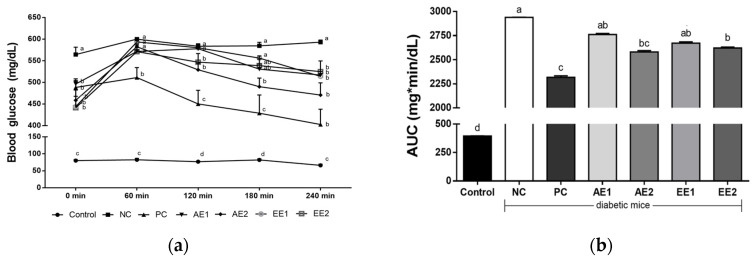
Effects of *A. hookeri* extracts on the OGTT of the diabetic mice. (**a**) Blood glucose changes, (**b**) area under the curve (AUC). Control: non-diabetic mice; NC: negative control; PC: positive control, metformin 150 mg/kg BW; AE1: aqueous extract 100 mg/kg BW; AE2: aqueous extract 200 mg/kg BW; EE1: ethanol extract 100 mg/kg BW; EE2: ethanol extract 200 mg/kg BW. AE and EE groups were treated with *A. hookeri* extracts for 8 weeks. Data are presented as the mean ± SEM (n = 8). ^a–d^ Mean values with different letters are significantly different at *p* < 0.05.

Oral glucose tolerance test (OGTT) experiments were performed to measure the effects of AH extracts on blood glucose tolerance in Type 2 diabetic mice by the 8th week (Figure 3a). Glucose (Sigma, MO, USA; 1 g/kg BW) was administered to each mouse, which fasted overnight, and blood was collected from their tail vein for 240 min. Before the glucose treatment, the NC group showed the highest blood glucose and the other diabetic mice showed significantly lower levels than that of the NC group. During the OGTT, the NC group consistently showed the highest blood glucose level and the other diabetic mice which were administered metformin or AH extracts showed lower values than those of the NC group. Diabetic mice administered high AH extracts (200 mg/kg BW) showed decreased blood glucose levels after 120 and 180 min of the glucose treatment. Blood glucose was significantly reduced in all experimental groups except the NC group at 240 min after oral glucose administration, and significantly decreased glucose levels were found in all the AH groups compared with that in the NC group.

The value of the area under the curve (AUC) was the highest in the NC group and decreased in all of the AH groups compared with the NC group (Figure 3b). A significant decrease in AUC was found in AE2 and EE2 groups compared with the NC group. 

### 2.5. Effects of A. hookeri Extracts on Glycated Hemoglobin (HbA1c), Insulin, Leptin, and Adiponectin Levels

**Figure 4:**
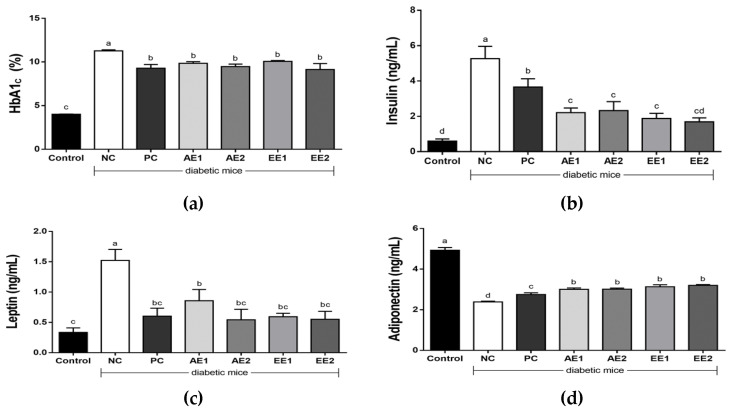
Effects of *A. hookeri* extracts on (**a**) blood HbA1c, (**b**) serum insulin, (**c**) leptin, and (**d**) adiponectin levels in the diabetic mice. Control: non-diabetic mice; NC: negative control; PC: positive control, metformin 150 mg/kg BW; AE1: aqueous extract 100 mg/kg BW; AE2: aqueous extract 200 mg/kg BW; EE1: ethanol extract 100 mg/kg BW; EE2: ethanol extract 200 mg/kg BW. AE and EE groups were treated with *A. hookeri* extracts for 8 weeks. Data are presented as the mean ± SEM (n = 8). ^a–d^ Mean values with different letters are significantly different at *p* < 0.05.

HbA1c shows the blood glucose condition in the last three months and is one of the most important indicators for diagnosing diabetes. The HbA1c level of diabetic mice was the highest in the NC group (10.9%) and its significant decrease was found in all the AH-treated groups, though there was no significant difference between the groups treated with AE and EE or the two doses (100 and 200 mg/kg BW) (Figure 4a). The results suggest that a dose of 100 mg/kg BW to diabetic mice can effectively lower the value of HbA1c. As shown in Figure 4b, the serum insulin level was significantly higher in the NC group (5.12 ± 0.18 ng/mL) than in the Control group (0.34 ± 0.07 ng/mL), and the values significantly decreased in the AH-treated mice compared with the NC group. Ethanol extract of AH was shown to effectively lower the serum insulin level without a significant difference between the EE1 and EE2 groups, treated with a dose of 100 or 200 mg/kg BW, respectively. 

Serum leptin level was also significantly higher in the NC group than in the Control group, but it was significantly decreased in all the AH aqueous or ethanol extract-administered groups (Figure 4c). In the NC group, the serum adiponectin level significantly decreased compared with that of the Control group. The adiponectin concentration was 2.8, 3.0, 3.0, 3.1, and 3.2 ng/mL in the PC, AE1, AE2, EE1, and EE2 groups, respectively. Both aqueous and ethanol AH groups showed significantly higher adiponectin levels compared with the NC group and the PC group treated with metformin (Figure 4d).

### 2.6. Effects of A. hookeri Extracts on Serum ALT, AST, BUN, and Cr Levels

**Figure 5:**
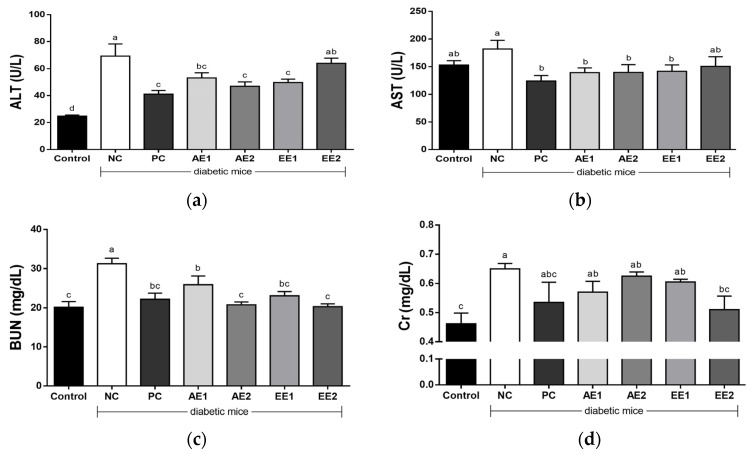
Effects of *A. hookeri* extracts on toxicological factors in the serum of the diabetic mice. (**a**) ALT, (**b**) AST, (**c**) BUN, and (**d**) Cr. Control: non-diabetic mice; NC: negative control; PC: positive control, metformin 150 mg/kg BW; AE1: aqueous extract 100 mg/kg BW; AE2: aqueous extract 200 mg/kg BW; EE1: ethanol extract 100 mg/kg BW; EE2: ethanol extract 200 mg/kg BW. AE and EE groups were treated with *A. hookeri* extracts for 8 weeks. Data are presented as the mean ± SEM (n = 8). ^a–d^ Mean values with different letters are significantly different at *p* < 0.05.

To evaluate whether AH extracts can improve toxicity in diabetic mice, the serum levels of hematological parameters such as alanine aminotransferase (ALT), aspartate aminotransferase (AST), blood urea nitrogen (BUN), and Cr were measured. Serum ALT, AST, BUN, and Cr values increased in the NC group compared with the Control group (Figure 5a–d). Their significant differences were found in the ALT, BUN, and Cr from the Control group (Figure 5a,c–d) and the higher levels in the NC group decreased in the AH extract-administered groups. AE and EE1 more effectively reduced ALT and AST levels than the EE2. AH extracts were comparable to or more highly effective than the metformin (PC group) in lowering BUN level, and the levels in AE2 and EE2 were recovered to those of the Control group (*p* > 0.05). Cr levels in the PC and AH groups decreased compared with the NC group, and the level of EE2 was also recovered to that of the Control group (Figure 5d). 

### 2.7. Effects of A. hookeri Extracts on Serum Lipid Profile

**Figure 6:**
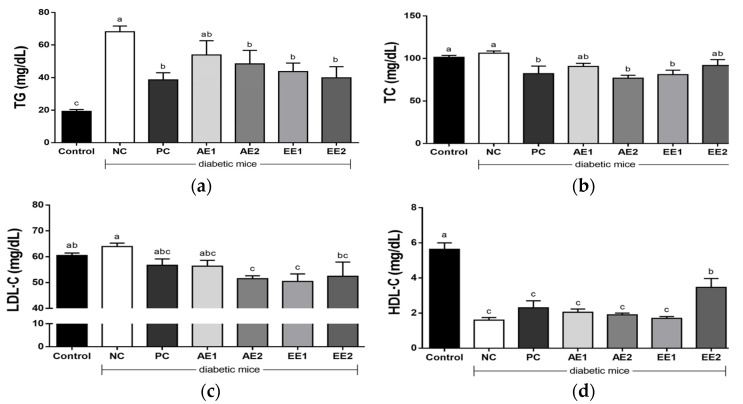
Effects of *A. hookeri* extracts on serum lipid profile in diabetic mice. (**a**) Triglyceride, (**b**) TC, (**c**) LDL-C, and (**d**) HDL-C. Control: non-diabetic mice; NC: negative control; PC: positive control, metformin 150 mg/kg BW; AE1: aqueous extract 100 mg/kg BW; AE2: aqueous extract 200 mg/kg BW; EE1: ethanol extract 100 mg/kg BW; EE2: ethanol extract 200 mg/kg BW. AE and EE groups were treated with *A. hookeri* extracts for 8 weeks. Data are presented as the mean ± SEM (n = 8). ^a–c^ Mean values with different letters are significantly different at *p* < 0.05.

As the glucose-related factors in the diabetic mice in this experiment, serum triglyceride (TG) and LDL-C levels were generally higher in the NC group compared with the Control group (Figure 6a–c). However, TG, TC, and LDL-C levels significantly decreased in the AE2 and EE1 groups while HDL-C was reduced in the NC group (Figure 6d). There was no significant difference in the serum TG, TC, and LDL-C levels between metformin- and AH-treated groups. The high serum lipid profile was markedly reduced, whereas HDL-C slightly increased in the AH extract-treated groups, and significant differences compared with the NC and PC groups were found in the EE2 group. 

### 2.8. Effects of A. hookeri Extracts on Histopathological Properties of Liver, Pancreas, and Kidney

**Figure 7:**
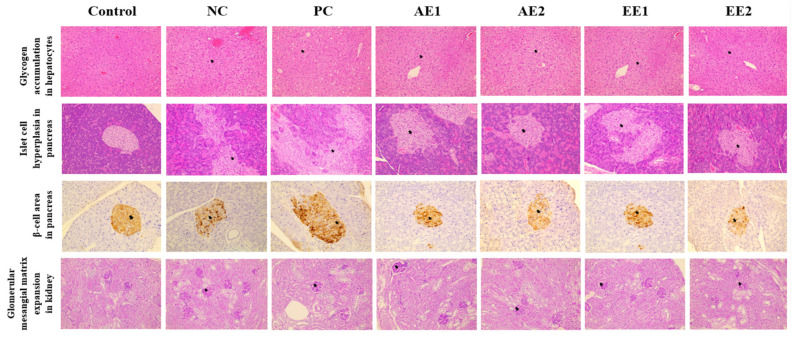
Effects of *A. hookeri* extracts on histopathological examination of the diabetic mice. Control: non-diabetic mice; NC: negative control; PC: positive control; AE1: aqueous extract 100 mg/kg BW; AE2: aqueous extract 200 mg/kg BW; EE1: ethanol extract 100 mg/kg BW; EE2: ethanol extract 200 mg/kg BW. AE and EE groups were treated with *Allium hookeri* extracts for 8 weeks. Data are presented as the mean ± SEM (n = 8).

The effects of AH extracts on the histopathological properties of the diabetic mice were evaluated and are shown in Figure 7. Glycogen accumulation was observed in all diabetic mice compared with the Control group. However, PC and EE groups showed decreased hepatic glycogen accumulation compared with the NC group. When hyperplasia of the pancreatic islet cell was evaluated in all experimental mice, hyperplasia was detected in all diabetic mice groups compared with the Control group. However, EE groups showed reduced hyperplasia compared with the NC group in a dose-dependent manner. 

**Figure 8:**
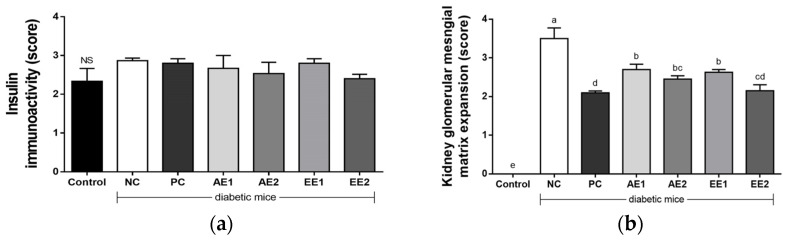
Effects of *A. hookeri* extracts on (**a**) insulin immunoactivity score in the pancreas and on (**b**) glomerular mesangial expansion score in the kidney of the diabetic mice. Control: non-diabetic mice; NC: negative control; PC: positive control; AE1: aqueous extract 100 mg/kg BW; AE2: aqueous extract 200 mg/kg BW; EE1: ethanol extract 100 mg/kg BW; EE2: ethanol extract 200 mg/kg BW. AE and EE groups were treated with *Allium hookeri* extracts for 8 weeks. Data are presented as the mean ± SEM (n = 8). ^NS^ Not significantly different among groups. ^a–e^ Different letters mean values are considered statistically significant at *p* < 0.05.

The β cell area of the pancreas was regular and clear in the Control group, and its shape in the NC group was irregular and unclear compared with that of the Control group. However, AE1 and EE1 groups showed improved shapes compared with the NC group. Immunohistochemical results show that insulin response increased in the diabetic mice compared with the Control group. PC and AH groups showed less immunoreactivity compared with the NC group (Figure 8a), although there was no significant difference. When the expansion of intravascular cell substrates of kidney was evaluated in the experimental mice, a significant increase was detected in the NC group compared with the Control group, and their decreases were recognized in the PC and AH groups (Figure 8b). More significant changes from the NC group were found in the EE groups. Thus, the histopathological results suggest that the conditions due to diabetes were improved in the EE groups rather than in the AE groups.

### 2.9. Effects of A. hookeri Extracts on the NK Activity in the Blood of Diabetic Mice

**Figure 9:**
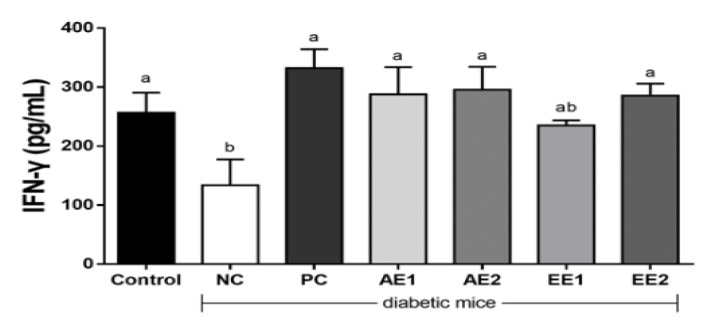
Effects of *A. hookeri* extracts on NK activity in the blood of diabetic mice. Control: non-diabetic mice; NC: negative control; PC: positive control, metformin 150 mg/kg BW; AE1: aqueous extract 100 mg/kg BW; AE2: aqueous extract 200 mg/kg BW; EE1: ethanol extract 100 mg/kg BW; EE2: ethanol extract 200 mg/kg BW. AE and EE groups were treated with *A. hookeri* extracts for 8 weeks. Data are presented as the mean ± SEM (n = 8). ^a,b^ Mean values with different letters are significantly different at *p* < 0.05.

Natural killer (NK) cell activity is one of the major factors indicating immune function, and a core element of innate immunity was evaluated in the diabetic mice. The IFN-γ concentration showing NK cell activity significantly decreased to 133.7 pg/mL in the NC group compared with 256.4 pg/mL in the Control group (Figure 9). However, IFN-γ concentrations increased in all the AH groups and were recovered to the level of the Control group. So, AH extracts are helpful for the recovery of NK cell activity decreased due to diabetes.

## 3. Discussion

AH is a plant of the genus *Allium* and contains special amino acids such as alliin, cycloalliin, and natural compounds such as volatile sulfur [27]. The sulfur-containing compounds of AH, allicin and alkyl thiosulfinate, lowered blood sugar and adipogenesis in diabetic models [24,25,26]. Alliin is converted into thiosulfinates by the enzyme alliinase when *Allium* vegetables are cut or crushed [28,29,30]. However, cycloalliin is not converted into thiosulfinate by alliinase and remains stable during processing [30]. Cycloalliin could be useful as a chemical and/or biological marker for AH.

This study investigated the effects of two AH extracts prepared by water or 50% ethanol on diabetic mice. The compounds are yellowish-brown powders with a unique flavor and without a strange taste or odor. The results show that the AH extracts decreased liver, kidney, and epididymal fat weight (% of BW), while it increased spleen weight in a dose-dependent manner (Table 1). The final body weight was lower in the AH groups than the NC group though there was no significant difference found among the T2D groups. Similar results were found in previous studies [24]. Kim, M.W. [31], Lee, J. [32], and Park et al. [33] also demonstrated that AH extract suppressed body and tissue weight in high-fat diet-fed models. 

Fasting blood glucose decreased in the AH groups and finally, a significant difference from the NC group was found in the EE groups, compared with the AE groups (Figure 2). Figure 3 shows the effects of *A. hookeri* extracts on OGTT in blood glucose changes (3a) and in AUC (3b). The AUC value increased in the NC group compared with the Control group, but AH extracts decreased it and a more significant difference from the NC group was found at a high dose (200 mg/kg BW) than at a low one (100 mg/kg BW). Kim et al. [24] reported that AH leaf or root reduced blood glucose and AUC compared with the NC group that was not treated with any AH extract. In particular, AH root significantly decreased these values in the study, and our results are similar to previous results. HbA1c shows the blood glucose condition for the last 3 months [24], and it decreased in all AH-treated groups, with significant differences in AH and PC groups compared with the NC group. Previous studies identified the anti-diabetic effects of AH in T2D, where Kim et al. [24] reported that the intake of diet supplemented with 1% and 3% extract of AH leaf or root reduced HbA1c concentration. Kim et al. [34] also found that the administration of methanol extract (400 and 800 mg/kg) from AH decreased the HbA1c concentration. The results of this study are similar to those of previous studies while low-level concentrations of the extracts such as 100 or 200 mg/kg BW were considered.

The serum insulin level increased in the NC group (5.3 ± 0.7 ng/mL) compared with the Control group (0.6 ± 0.1 ng/mL) due to hyperinsulinemia, as shown in the T2D model (Figure 4b). The high insulin level in the NC group significantly decreased in the PC and AH groups. A more significant reduction was found in the EE groups in a dose-dependent manner and the level of the EE2 group was recovered to that of the Control group. Insulin is secreted to enhance the glycolysis process and lower glucose levels when glucose concentration is high [35]. In this study, blood glucose concentration was reduced in the AE and EE groups and insulin value also decreased in these groups. Therefore, these results imply that AH ethanol extract may lower the value of glycated hemoglobin and has the ability to improve insulin sensitivity. Leptin is known to decrease food intake, facilitate glucose utilization, and improve insulin sensitivity in the maintenance of metabolic balance [35]. A significantly increased blood leptin level was observed in the NC group (1.5 ± 0.2 ng/mL) compared with the Control group (0.3 ± 0.1 ng/mL). However, all the AH groups and the PC group administered metformin showed decreased values compared with the NC group (Figure 4c), which exhibited increased value due to the abnormal metabolic condition of glucose in the T2D model. More effective reductions in the serum leptin levels were found in the EE groups than in the AE1 group, and they were recovered to those of the Control group. While the adiponectin level of the NC group was significantly lower than that of the Control group, the AH groups and the PC group showed significant increases compared with the NC group (Figure 4d). Adiponectin has potent effects on the glucose metabolism in the liver and improves glucose uptake, and leptin and adiponectin have an indirect and inverse correlation with insulin resistance [35]. In this study, AE and EE induced the increase in adiponectin concentration, while they reduced the leptin concentration. This may be explained by the fact that they enhance adiponectin secretion and effectively control blood glucose, insulin, and leptin levels. Given these results, AH could be presumably used as a good natural anti-diabetic source.

Furthermore, AH extracts reduced the toxicity found in the diabetic mice by reducing the levels of hematological parameters such as serum ALT, AST, BUN, and Cr, which increased in the NC group compared with the Control group (Figure 5a–d). Lee, J. [32] identified that the administration of a high-fat diet with 5% AH induced a decrease in ALT value, and the 3% AH group showed a reduction in AST value. ALT and AST are mainly used in the evaluation of hepatic damage and the increase in ALT and AST may be due to liver dysfunction [36]. Hence, AH aqueous or ethanol extracts were considered for the recovery of liver injury. The measurement of urea and Cr can be accomplished with easily available tests for the detection and prevention of diabetic kidney disease [37]. In this study, there was a decrease in BUN and Cr concentration due to the administration of AE and EE. *A. hookeri* may be used to relieve BUN and Cr values in diabetes patients and help them to recover from diabetic kidney disorder.

It has been reported that diabetes causes dyslipidemia, such as obesity and related hyperlipidemia. Symptoms of hyperlipidemia include the increased synthesis of TG, TC, LDL-C, and decreased HDL-C. The effects of leptin and adiponectin were indirect and showed an inverse correlation with insulin resistance, triglycerides, and LDL-C [36]. In this study, there was a reduction in TG, TC, and LDL-C, and an increase in HDL-C value in the AE and EE groups (Figure 6). We found that ethanol extract more effectively improved the blood cholesterol conditions. Kim et al. [34] reported that diabetic mice supplemented with 5% and 10% AH root induced an increase in HDL-C and a reduction in TG and AST. Previous studies [32,33] showed that AH root decreased TG, TC, and LDL-C levels, while they increased HDL-C. 

It has been reported that toxicological factors in blood and tissues increase in diabetic mice due to the inappropriate working of the liver and kidneys [37]. The mechanism can be partially explained by the effective histopathological changes, and an improvement in the glycogen accumulation in the liver, hyperplasia in the pancreatic islet cell, irregular shape of the β-cell area, and the increased expansion of the intravascular cell substrates of the kidney were detected in the diabetic NC group (Figure 7 and Figure 8). However, PC and EE groups showed decreased hepatic glycogen accumulation compared with the NC group. EE groups showed reduced hyperplasia of the pancreatic islet cell compared with the NC group in a dose-dependent manner. The β cell area of the pancreas, which was irregular in the NC group, was improved in the AE1 and EE1 groups compared with the NC group. The increase in the insulin response in the diabetic mice compared with the Control group tended to decrease in the EE2 group (Figure 8a). The significant expansion of the intravascular cell substrates of kidney in the NC group compared with the Control group decreased in the PC and AH groups. A more effective reduction from the NC group was further detected in the EE groups. Taking this account, the conditions due to diabetes were significantly improved in the EE groups rather than in the AE groups. 

T2D drives immune dysfunction and infection development [14]. The relationship between NK cell activity and glucose control in patients with Type 2 diabetes and prediabetes was previously reported [38]. Individuals with T2D are physiologically frail and have an increased risk of infections and mortality from sepsis. Immune-modulatory therapies have been utilized in other chronic inflammatory diseases, and are used to control the chronically affected immune pathways in T2D patients. Immune function is especially important in diabetic patients. So, in this study, NK cell activity, which is a representative immune relative factor, was evaluated in the experimental animals. AH extracts increased IFN-γ production, showing improved NK cell activity, thereby suggesting their potential to immune function recovery against diabetes (Figure 9). 

## 4. Materials and Methods

### 4.1. Preparation of Extract Samples

AH roots were obtained from a farm at the Sunchang area in Jeonbuk (South Korea) and were authenticated by the National Institute of Agricultural Sciences. After a multiple-step cleaning process and drying at 50 °C for 2 days, 5 kg of dried AH roots was extracted twice with 10 times volume of 50% *v/v* ethanol using the extracting and concentration system (HS Tech., Seongnam, Korea) at 40 °C for 8 h for extraction, and at 65 °C and 650 mH for concentration (EE). For the AE, 5 kg of dried AH roots was extracted twice with 10 times volume of hot water (50 L) at 90 °C for 8 h, filtered through 25 µm, and concentrated at 65 °C and 650 mH using the extracting and concentration system (HS Tech.). The AH extracts were then frozen and lyophilized (PVTFD 300R, Ilsin Lab, Yangju, Korea). The final extracts were EE 1.89 (37.8%) and AE 1.93 kg (38.6%). The samples (RDAAHR-E01, RDAAHR-A01) were stored at −70 °C in the Department of Agricultural Food Resources, National Institute of Agricultural Sciences, Rural Development Administration.

### 4.2. Measuring Cycloalliin Concentration

To analyze the cycloalliin, sample (or cycloalliin hydrochloride monohydrate as a standard, Fujifilm Wako Pure Chemical Co., Osaka, Japan) was dissolved in methanol at 0.1 g/mL. Agilent 6410 Triple Quad LC/MS (Agilent Technologies, Santa Clara, CA, USA) coupled to a MS QQQ mass spectrometer equipped with an electrospray ionization sources (Agilent Technologies) was used. Chromatographic separations were performed on a reversed phase C18 with polar end-capping (15 mm × 2 mm, Synergi^TM^ 4 μm Hydro-RP 80Å; phenomenex, Torrance, CA, USA). The operating temperature was set at 30 °C and the flow rate was 0.2 mL/min. Mobile phase A and B consisted of 0.1% formic acid in water and 0.1% formic acid in acetonitrile, respectively. The gradient system was as follows: 0 min, 5% B; 1 min, 5% B; 11 min, 100% B; 12 min, 100% B; 15 min, 5% B; and 20 min, 5% B. The mass spectrometer was operated using MS QQQ Mass Spectrometer for electrospray ionization (ESI). The general settings used were as follows: gas temperature, 300 °C; gas flow, 11 L/min; nebulizer, 15 psi; capillary, 4000 V. Regression equation (y) is 233.26x + 259.29 and correlation coefficient (R2) is 1.

### 4.3. Animals and Diets

Male C57BL/J-*db/db* mice (6 weeks old) and male C57BL/J-m+/*db* mice (6 weeks old) were purchased from Central Lab, Animal Inc. (Seoul, Korea). During the experimental period, all the mice had free access to pelleted feeds and water. The mice were kept in a room with 23 ± 2 °C, 50 ± 10% relative humidity, and a 12 h light/12 h dark cycle. After a 1-week acclimatization period, the mice were randomly divided into 7 groups (8 mice in each group): (1) non-diabetes group (Control, C57BL/J-m+/db mice), (2) diabetes control group (negative control; NC, C57BL/J-*db/db*), (3) positive control group (PC, C57BL/J-*db/db*, 150 mg metformin/kg BW), (4) AE1 group with low dose of extract (C57BL/J-*db/db*, 100 mg AE/kg BW), (5) AE2 group with high dose of extract (C57BL/J-*db/db*, 200 mg AE/kg BW), (6) EE1 group with low dose of extract (C57BL/J-*db/db*, 100 mg EE/kg BW), and (7) EE2 group with high dose of extract (C57BL/J-*db/db*, 200 mg EE/kg BW). Oral administration for each group was performed for 8 weeks. The condition of the experimental animals was monitored every day, and food intake and body weight were recorded once a week. Permission for the animal experiments was granted by the Animal Experimentation Ethics Committee (NAS-202106) of the National Institute of Agricultural Sciences, Rural Development Administration, and the ethical regulations were followed.

### 4.4. Measuring Blood Glucose and Conducting Oral Glucose Tolerance Test (OGTT)

Fasting blood glucose was measured from the tail vein using a glucometer (Accu-Check Active, Roche Diagnostics GmbH, Mannheim, Germany) after starvation for 12 h every 2 weeks. OGTT was conducted after 12 h fasting on the 8th week of treatment. Blood was collected from the tail vein of mice at 30, 60, 90, 120, 180, and 240 min after oral administration of glucose at 1.5 g/kg BW (Sigma). The AUC was measured by trapezoidal approximation of blood glucose levels. Blood glucose at x min was defined as BG (x), and AUC was calculated as follows:

AUC (mg*h/dL) = {BG (0) + BG (60)}/2 + {BG (60) + BG (120)}/2 + {BG (120) + BG (180)}/2 + {BG (180) + BG (240)}/2

### 4.5. Blood Biochemical Analysis 

After the experiment period was over, the mice were sacrificed under anesthesia (CO_2_) after measuring their body weight, and blood was collected from the abdominal vena cava. HbA1c concentration was measured from the whole blood by the boronate affinity-based method using EasyA1c (Osang Healthcare, Anyang, Korea). For more blood chemical analysis, the blood was centrifuged at 2000 rpm for 15 min to separate serum. Serum insulin concentration was measured using a Mouse Insulin ELISA kit (AKRIN-011T, Fujifilm Wako Shibayagi Co., Gunma, Japan). Biotinylated anti-insulin antibody and standard or sample were incubated for 2 h in monoclonal anti-insulin-coated wells. After washing the cultured wells, horseradish peroxidase (HRP)-conjugated streptavidin was added and incubated for 30 min. After discarding the reaction mixture and washing the plate, chromogen (TMB) was added, the sample was reacted for 30 min, and the reaction was stopped by adding a stop solution. The absorbance was measured at 450 nm using a plate reader (Microplate reader, TECAN, Männedorf, Switzerland). The serum adiponectin concentration was measured using a mouse Adiponectin ELISA kit (MRP300, R&D system Inc., Minneapolis, MN, USA). Standards and samples were placed in antibody-coated wells and incubated for 3 h at room temperature. After incubation and washing, Adiponectin Conjugate was added and the sample was incubated for 1 h, then washed. Substrate solution was added to the well and incubated for 30 min at room temperature. After that, stop solution was added and the absorbance measured at 450 nm using a plate reader (Microplate reader, TECAN) within 30 min. The serum leptin concentration was measured using a mouse Leptin ELISA kit (CSB-E04650m, Cusabio, Houston, TX, USA). The standards and samples were placed into the leptin antibody-encoded wells and incubated for 2 h at 37 °C. After that, Biotin antibody was added to the wells and incubated for 1 h at 37 °C. Each well was washed, HRP-avidin was added, and the sample was incubated at 37 °C for 1 h. After washing, TMB substrate was added to each well and incubated at 37 °C for 30 min. Finally, stop solution was added and the absorbance measured using a plate reader (Microplate reader, TECAN) within 5 min at 450 nm. Lipid profile (triglyceride, TC, LDL-C, and HDL-C levels) and toxicological factors (ALT, AST, BUN, and Cr) were measured using a blood biochemical analyzer (7180, HITACHI, Tokyo, Japan). NK cell activity in the blood was evaluated using the NK activity kit (ATGen, Seongnam, Korea), which is an IFN-γ quantitation assay for plasma samples collected and prepared with the NK cell activating agent. Absorbance was measured at 450 nm, and the amount of IFN-γ released by the NK cells was finally quantitated by comparison to an IFN-γ standard curve.

### 4.6. Histopathological and Immunohistochemical Investigations

After blood collection, the mice were euthanized by cervical dislocation; organs or tissues such as liver, kidney, epididymal fat, and spleen of mice were excised; and the weight of each organ was measured. Liver, pancreas and kidney were fixed with 10% formalin solution at room temperature for 24 h. The fixed tissue was embedded in paraffin and cut into 3 μm sections using a Bond Polymer Intense Detection system (Vision BioSystems, Melbourne, Australia). The sectioned tissue was pre-treated, double-stained with hematoxylin and eosin, and subjected to Periodic Acid-Schiff (PAS) reaction, followed by dehydration. After sealing the cover glass with a synthetic resin encapsulant, it was observed under an optical microscope at 200× magnification and photographed with a camera attached to a microscope.

### 4.7. Statistical Analysis

Statistical analysis was performed using the mean ± SEM by one-way ANOVA (one-way analysis of variance) using the SPSS (Statistical Package for the Science ver. 24, IBM Corp, Armonk, NY, USA). A probability level of *p* < 0.05 was accepted statistically.

## 5. Conclusions

In this study, AH extracts improved glucose metabolism by controlling fasting blood glucose, glucose tolerance, and insulin activity, and prevented the occurrence of diabetic-related symptoms without any toxicity. EE was identified to be more effective in reducing high blood glucose and lipid levels than AE, and is interestingly comparable to metformin in some biomarkers. It is suggested that EE may be used as a functional supplement for the prevention of diabetic complications in the Type 2 diabetic model.

## Figures and Tables

**Table 1:** Effects of *A. hookeri* extracts on body and organ/tissue weights of the diabetic mice.

	Control	NC	PC	AE1	AE2	EE1	EE2
Initial body weight (g)	21.87 ± 0.82 ^b^	32.53 ± 5.29 ^a^	33.63 ± 4.16 ^a^	33.42 ± 3.96 ^a^	33.93 ± 4.26 ^a^	31.73 ± 3.96 ^a^	33.65 ± 4.25 ^a^
Final body weight (g)	27.65 ± 0.31 ^b^	33.55 ± 0.82 ^a^	32.62 ± 3.63 ^a^	32.82 ± 3.82 ^a^	29.35 ± 3.41 ^a^	32.84 ± 3.78 ^a^	29.86 ± 3.21 ^a^
Tissue weight (% of BW)							
Liver	3.78 ± 0.08 ^c^	6.28 ± 0.21 ^a^	5.40 ± 0.16 ^b^	5.25 ± 0.06 ^b^	5.38 ± 0.21 ^b^	5.42 ± 0.11 ^b^	5.59 ± 0.14 ^b^
Kidneys	0.98 ± 0.08 ^c^	1.44 ± 0.09 ^a^	1.01 ± 0.04 ^c^	1.11 ± 0.03 ^bc^	1.10 ± 0.07 ^c^	1.24 ± 0.05 ^a^^b^	1.27 ± 0.03 ^ab^
Epididymal fat	2.22 ± 0.10 ^d^	5.39 ± 0.15 ^a^	5.09 ± 0.16 ^ab^	5.10 ± 0.10 ^ab^	4.79 ± 0.17 ^bc^	4.91 ± 0.14 ^ab^	4.43 ± 0.22 ^c^
Spleen	0.27 ± 0.02 ^a^	0.10 ± 0.01 ^c^	0.13 ± 0.01 ^bc^	0.12 ± 0.01 ^bc^	0.16 ± 0.02 ^b^	0.15 ± 0.03 ^b^	0.17 ± 0.02 ^b^

Control: non-diabetic normal mice; NC: negative control; PC: positive control, metformin 150 mg/kg BW; AE1: aqueous extract 100 mg/kg BW; AE2: aqueous extract 200 mg/kg BW; EE1: ethanol extract 100 mg/kg BW; EE2: ethanol extract 200 mg/kg BW. AE and EE groups were treated with *A. hookeri* extracts for 8 weeks. Data are presented as the mean ± SEM (n = 8). ^a–c^ Mean values with different letters are significantly different at *p* < 0.05.

## Data Availability

Data is contained within the article.
